# Trajectories of Postoperative Depressive Symptoms in Older Patients Undergoing Major Surgery

**DOI:** 10.1001/jamanetworkopen.2023.54154

**Published:** 2024-01-31

**Authors:** Irena Cenzer, Sharon K. Inouye, Patrick J. Raue, Christina Keny, Zara Cooper, Victoria L. Tang

**Affiliations:** 1 Division of Geriatrics, University of California, San Francisco; 2 Department of Medicine, Beth Israel Deaconess Medical Center, Harvard Medical School, Boston, Massachusetts; 3 Marcus Institute for Aging Research, Hebrew SeniorLife, Boston, Massachusetts; 4 Department of Psychiatry & Behavioral Sciences, University of Washington, Seattle; 5 Division of Geriatrics, Department of Medicine, Veterans Affairs Medical Center, San Francisco, California; 6 School of Nursing, University of California, San Francisco; 7 Center for Surgery and Public Health, Brigham and Women’s Hospital, Boston, Massachusetts; 8 Department of Surgery, Brigham and Women’s Hospital, Boston, Massachusetts; 9 Division of Hospital Medicine, Department of Medicine, Veterans Affairs Medical Center, San Francisco, California

## Abstract

This cohort study examines the trajectories of postoperative depressive symptoms in older patients undergoing major surgery and the differences in patient characteristics between the trajectory groups.

## Introduction

More than one-half of older adults undergoing major surgery report preoperative depressive symptoms.^1^ Depressive symptoms have been identified as a factor associated with functional decline, yet depression is an understudied and important patient-centered outcome.^2^ This cohort study (1) describes the trajectories of depressive symptoms for 18 months after surgery and (2) examines differences in patient characteristics between different trajectory groups.

## Methods

The Successful Aging after Elective Surgery (SAGES) study is a prospective cohort study of adults at least 70 years old undergoing major elective surgery (ie, orthopedic, gastrointestinal, or vascular),^3^ with an anticipated length of stay of at least 3 days at 1 of 2 academic medical centers. Both study hospitals’ institutional review boards approved the study. This study followed the STROBE reporting guideline. Patients who completed the 15-item Geriatric Depression Scale (GDS) before surgery and at 6, 12, and 18 months postoperatively were included. Patients provided written informed consent.

Baseline sociodemographic, functional, cognitive, and psychosocial assessments were collected from patient interviews preoperatively. The main outcome was number of depressive symptoms. Trajectory mixture models identified the optimal number of clusters of individuals following similar trajectories of depressive symptoms. The optimal number of clusters was determined using the lowest values of bayesian information criterion and Akaike information criterion, indicative of better model fit, and the highest entropy, reflecting better classification accuracy. Statistical tests (χ^2^ tests) were significant if 2-sided *P* < .05. Analyses used Stata/MP statistical software version 18.0 (StataCorp)^4^ and were conducted from June 2022 to October 2023.

## Results

A total of 560 patients met eligibility criteria and were enrolled between June 8, 2010, and August 8, 2013. Our study included 487 patients who completed the GDS (mean [SD] age, of 76.5 [4.9] years; 278 [57.1%] women; 454 [93.2%] White). Of the 73 excluded, 11 died, and 62 did not complete the assessment or dropped out.

The mean (SD) preoperative GDS score was 2.4 (2.5) (median [IQR] score, 2 [1-3]). The continuous GDS scale improved 0.46 points in the first 6 months, but did not change in the 2 subsequent 6-month periods (change, −0.09 and 0.03 points, respectively).

We identified 3 depressive symptom trajectories: (1) sustained low (150 patients [31%]), (2) sustained moderate (290 patients [59%]), and (3) sustained high (47 patients [10%]) (Figure). The first 2 groups showed a statistically significant but small improvement immediately after surgery, followed by no significant changes over the next 12 months. The third group showed no significant changes over 18 months.

**Figure.**
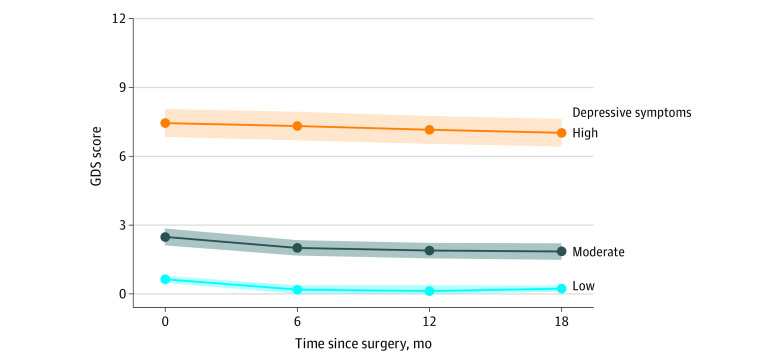
Trajectories of Depressive Symptoms for 18 Months After Surgery GDS indicates Geriatric Depression Scale.

Comparisons of the 3 groups are shown in the Table. Higher sustained depressive symptoms were associated with age older than 80 years (16% vs 27% vs 38%), activities of daily living dependence (2% vs 8% vs 19%), instrumental activities of daily living dependence (13% vs 29% vs 53%), and higher baseline depressive burden (0% vs 6% vs 81%).

**Table.** Baseline Characteristics of Participants by Depressive Symptom Trajectory

Characteristics	Participants, No. (%)			*P* value^a^
	Low depressive symptoms (n = 150 [31%])	Moderate depressive symptoms (n = 290 [59%])	High depressive symptoms (n = 47 [10%])	
Age >80 y	24 (16)	78 (27)	18 (38)	.003
Sex				
Women	82 (55)	167 (58)	29 (62)	.67
Men	68 (45)	123 (42)	18 (38)	
Duration of education ≥12 y	119 (79)	203 (70)	29 (62)	.03
Married or partnered	93 (62)	174 (60)	26 (55)	.71
Lives alone	44 (29)	84 (29)	17 (36)	.60
Charlson Comorbidity Index score				
0	82 (55)	135 (47)	14 (30)	.06
1	32 (21)	71 (24)	15 (32)	
≥2	36 (24)	84 (29)	18 (38)	
Surgery type				
Orthopedic	123 (82)	235 (81)	39 (83)	.09
Vascular	3 (2)	22 (8)	3 (6)	
Gastrointestinal	24 (16)	33 (11)	5 (11)	
Modified Mini-Mental State Examination score <85^b^	5 (3)	15 (5)	5 (11)	.12
≥1 ADL dependence^c^	3 (2)	24 (8)	9 (19)	<.001
≥1 IADL dependence^d^	19 (13)	84 (29)	25 (53)	<.001
Baseline Geriatric Depression Scale score				
No depressive burden	130 (87)	101 (35)	2 (4)	<.001
Mild depressive burden	20 (13)	172 (59)	7 (15)	
High depressive burden^e^	0	17 (6)	38 (81)	

Abbreviations: ADL, activities of daily living; IADL, instrumental activities of daily living.

^a^ *P* values are calculated using the χ^2^ test.

^b^ The Modified Mini-Mental State Examination was designed to assess general cognitive ability in older adults (score 71-84 indicates cognitively impaired, and score 85-100 indicates cognitively intact).

^c^ ADLs are measured on the Katz Scale by these activities: bathing, grooming, dressing, feeding, transfers (bed to chair), toileting, and walking. ADL independent is defined by not needing help in completing any of the ADL activities. ADL dependent is defined by needing help in 1 or more of the ADL activities.

^d^ IADLs are measured by the Lawton Scale by these activities: shopping, preparing meals, managing medications, using the telephone, housework, laundry, driving or using public transportation, and managing finances.

^e^ Categorized by the number of depressive symptoms, with 0 to 1 indicating no depressive burden, 2 to 5 indicating mild depressive burden, and 6 to 15 indicating high depressive burden.

## Discussion

This cohort study found no significant changes in depressive symptom burden among older adults up to 18 months after surgery. Corroborating findings in other studies,^5^ we found that postoperative depressive symptoms were significantly associated with preoperative depression and with functional dependence. Physical functioning after surgery, which is negatively impacted by depressive symptoms, is of paramount importance to older patients. Thus, the clinical importance of our findings is that depressive symptoms are a potentially modifiable factor to improve patient-centered postoperative outcomes and health trajectories, and the surgical event represents an opportune time to screen for and intervene on this important risk factor.

A potential limitation to our study is the sample size of the cohort; therefore, a multivariable model is beyond the scope of this study. However, our primary goal for this study was to begin describing and highlighting an important and understudied postoperative patient-centered outcome. Further studies will be needed to address the multivariable modeling of the differing trajectories. Our results underscore opportunities for screening older surgical patients and providing early intervention focused on both depressive symptom burden treatment and enhancing physical functioning.

## Notes

Supplement.
Data Sharing Statement
