# Supplementary material for: Trajectories of Postoperative Depressive Symptoms in Older Patients Undergoing Major Surgery
Source: JAMA Netw Open. 2024 Jan 31;7(1):e2354154. doi: 10.1001/jamanetworkopen.2023.54154 (PMC10831558; doi:10.1001/jamanetworkopen.2023.54154)
Supplement: Supplement. — Data Sharing Statement [file jamanetwopen-e2354154-s001.pdf]

# Data Sharing Statement

Cenzer. Trajectories of Postoperative Depressive Symptoms in Older Patients Undergoing Major Surgery. *JAMA Netw Open*. Published January 31, 2024.
doi:10.1001/jamanetworkopen.2023.54154

**Data**
**Data available:** No

**Additional Information**
**Explanation for why data not available:** This data was collected by and belongs to Harvard Medical School. We have no premission to share the data.
